# Radiomics analysis for distinctive identification of COVID-19 pulmonary nodules from other benign and malignant counterparts

**DOI:** 10.1038/s41598-024-57899-x

**Published:** 2024-03-25

**Authors:** Minmini Selvam, Anupama Chandrasekharan, Abjasree Sadanandan, Vikas K. Anand, Sidharth Ramesh, Arunan Murali, Ganapathy Krishnamurthi

**Affiliations:** 1 Department of Radiology and Imaging Sciences, Sri Ramachandra Institute of Higher Education and Research, Porur, Chennai, 600 116 India; 2 Department of Engineering Design, Indian Institute of Technology-Madras, Chennai, 600 036 India

**Keywords:** Radiomics, Lung, Nodules, COVID-19, Machine learning, Classifiers, Cancer, Medical research

## Abstract

This observational study investigated the potential of radiomics as a non-invasive adjunct to CT in distinguishing COVID-19 lung nodules from other benign and malignant lung nodules. Lesion segmentation, feature extraction, and machine learning algorithms, including decision tree, support vector machine, random forest, feed-forward neural network, and discriminant analysis, were employed in the radiomics workflow. Key features such as Idmn, skewness, and long-run low grey level emphasis were identified as crucial in differentiation. The model demonstrated an accuracy of 83% in distinguishing COVID-19 from other benign nodules and 88% from malignant nodules. This study concludes that radiomics, through machine learning, serves as a valuable tool for non-invasive discrimination between COVID-19 and other benign and malignant lung nodules. The findings suggest the potential complementary role of radiomics in patients with COVID-19 pneumonia exhibiting lung nodules and suspicion of concurrent lung pathologies. The clinical relevance lies in the utilization of radiomics analysis for feature extraction and classification, contributing to the enhanced differentiation of lung nodules, particularly in the context of COVID-19.

## Introduction

The corona virus disease of 2019 (COVID-19) is an infection caused by a novel SARS-CoV-2 virus (corona virus). Due to the rapid community spread of the virus, on January 30, 2020, the World Health Organization (WHO) declared the outbreak a Public Health Emergency of International Concern and on 11 March 2020 as a pandemic^1^. As of 3rd March 2024, there have been 774,834,251 confirmed cases of COVID-19 including 7,037,007 deaths reported to WHO, and a total of 13.59 billion doses of COVID-19 vaccines have been administered globally^2^. The identification of patients infected by COVID plays a crucial role in controlling the disease process and isolation of patients, thereby preventing the further spread of infection. A definitive diagnosis of COVID requires a positive reverse transcription-polymerase chain reaction (RT–PCR) test and lateral flow immunochromatographic assays which are immunoassay-based techniques that have also been performed in detecting SARS-COV-2 antigens^3^. Chest CT scans in patients with COVID-19 accurately depict the various features and extent of involvement, monitor the clinical course, and evaluate the disease severity, progression, and complications^4^. The primary findings on CT have been reported as ground-glass opacities (GGO), crazy paving appearance (GGOs and inter-/intra-lobular septal thickening), air space consolidation, broncho vascular thickening, and traction bronchiectasis^5–8^. The ground-glass and/or consolidative opacities are usually bilateral, peripheral, and basal in distribution^9^. Less commonly seen CT findings include mediastinal lymphadenopathy, pleural effusions, lung nodules, reverse halo sign, cavitation, pneumothorax, and pneumomediastinum^10^.

Studies have also shown that at 3 months after acute infection, a subset of patients will have CT abnormalities that include GGO and subpleural bands with concomitant pulmonary function abnormalities. At 6 months after acute infection, some patients have persistent CT changes including residual GGOs seen in the early recovery phase and the persistence or development of changes suggestive of fibrosis and reticulation with or without parenchymal distortion^11,12^. Broadly speaking, the etiology of lung nodules is varied and includes benign non-neoplastic causes like infections (granulomas, round pneumonia, septic emboli), benign non-infectious causes (amyloidoma, subpleural lymph nodules, rheumatoid nodules, Wegner’s granulomatosis), benign tumors (hamartoma, carcinoid, neurofibroma, etc.) and malignant neoplasms (primary lung carcinoma, lymphoma, and metastasis)^13^. Lung nodules contribute to a small percentage (3−13 %) of CT manifestations of acute coronavirus infection and may occasionally be seen along with other pulmonary findings^14^.

Radiomics has recently emerged as a promising tool in the field of medical imaging. It uses high-throughput extraction of a large number of quantitative features from radiological images and converts these images into mineable data that can be analyzed. This data then contributes to clinical decision support for improved diagnostic, prognostic, and predictive accuracy^15^. The application of radiomics to the thorax has so far been majorly focused on lung cancer. Recently studies have been performed on radiomics in COVID-19 which showed that the machine learning-based CT radiomics models may accurately classify COVID-19, helping clinicians and radiologists to identify COVID-19-positive cases, distinguish COVID-19 pneumonia vs. other pneumonia, and distinction the severity of COVID-19 pneumonia^16^. However, the role of radiomics in COVID-19 pulmonary models has not yet been described. We sought to study the radiomics features of COVID-19 lung nodules and to compare these findings with radiomics features of other benign non-COVID-19 lung nodules and malignant lung nodules. In summary, radiomics can be used as a powerful tool in modern medicine, as an adjunct to CT and/or PET–CT images, in further evaluation of lung nodules/mass lesions to help in clinical decision support and to improve diagnostic, predictive, and prognostic accuracy. Thus, radiomics has an important role in cancer staging, and diagnosis and aids in precision oncology. A preliminary account of this work is presented elsewhere^17^.

## Materials and methods

**Figure 1:**
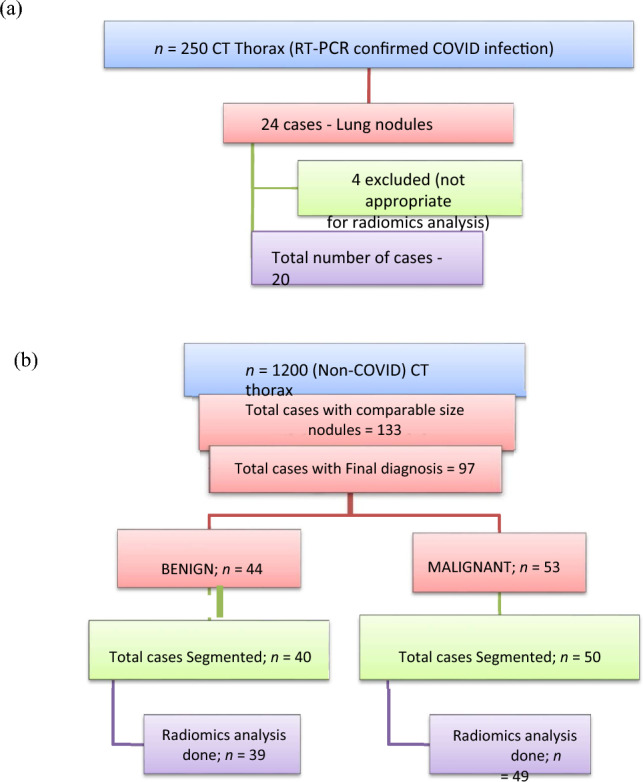
(**a**) Flow diagram of the study of COVID-19-infected cases. (**b**) Flow diagram of the study of non-COVID-19-infected cases.

A cross-sectional observational study was performed in our department after obtaining prior approval from the Institute Ethics Committee. We initially reviewed CT thorax scans of 250 patients with RT-PCR-positive COVID-19 infection over one year as detailed in Fig. 1a. Lung nodules were present in 24 patients with COVID-19 infection of which four patients were eliminated from our study as their CT images were not suitable for radiomics analysis. Only patients in whom no other synchronous lung pathology was identified were included. The imaging features of COVID-19 lung nodules were studied in the remaining 20 patients and were assessed for size, type, margins, location, and the lobe and segment involved, Digital imaging and communications in medicine (DICOM) images of these 20 patients were thereafter subjected to segmentation analysis and radiomics post-processing.

We exclusively examined patients with COVID pneumonia who exhibited solid pulmonary nodules only, totaling 20 cases, to ensure similarity with benign and malignant nodules. Our study involved individuals who underwent chest CT scans between the 3rd and 6th day after the onset of symptoms (1st week of the disease) and who tested positive for COVID-19 infection through RT-PCR. Along with these pulmonary nodules, the other common CT findings in these patients typically included ground glass opacities, crazy paving, and consolidation. However, our focus for radiomics analysis was solely on these pulmonary nodules.

We reviewed CT thorax of 1200 non-COVID-19 patients as shown in Fig. 1b. Lung nodules of compatible size were present in 133 cases. The final diagnosis was available in 97 patients, of whom 44 were benign and 53 were malignant nodules. The benign lesions were diagnosed based on histopathological diagnosis or correlation with clinical features and follow-up as per the Fleisher’s Society guidelines^18^. The final diagnosis in primary malignant lesions was arrived at based on histopathological diagnosis, and in metastasis based on the histopathological diagnosis of the lung nodule or primary tumor. The DICOM images of 40 benign nodules and 50 malignant nodules were subjected to segmentation analysis. Radiomics post-processing was done in 39 benign nodules and 49 malignant nodules. Radiomics analysis was not feasible in two patients. Subsequently, the radiomics texture analysis of COVID-19 lung nodules was compared separately with each radiomics analysis of benign non-COVID-19 benign lung nodules and malignant lung nodules.

**Figure 2:**
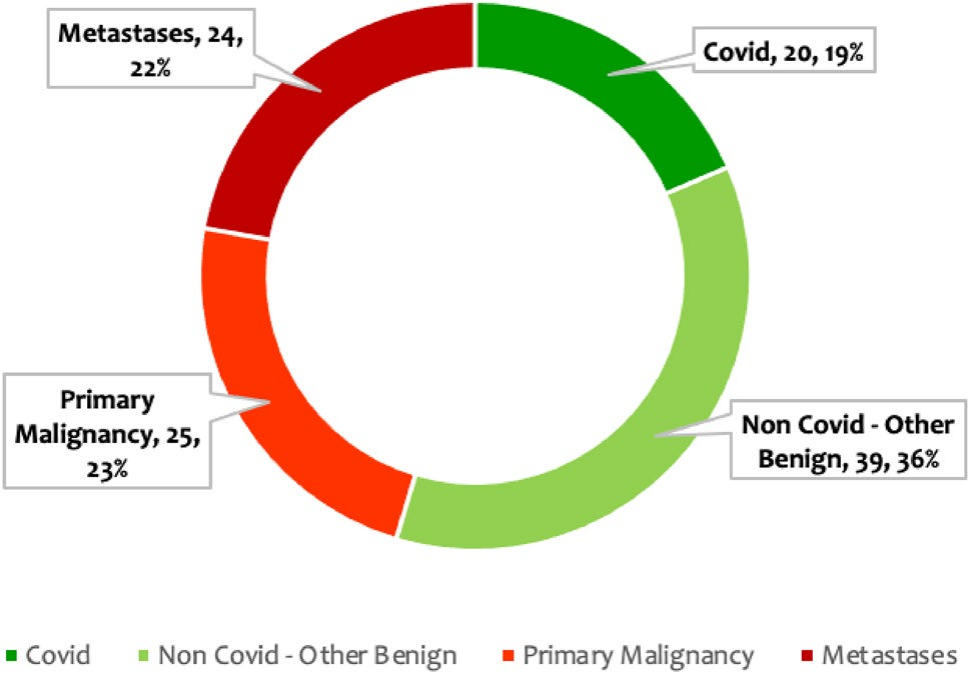
Case distribution of the lung nodules included in the study.

The distribution of cases included in the final radiomics analysis included, *n* = 24 (22%) metastatic pulmonary nodules, *n* = 25 (23%) primary malignancies, *n* = 39 (36%) non-COVID benign nodules, and *n* = 20 (19%) COVID-related nodules (Fig. 2). The final diagnosis was available in all these nodules. *n* = 39 of the pulmonary nodules were found to be other benign, while *n* = 49 were malignant. The benign lesions were diagnosed based on histopathological diagnosis or correlation with clinical features and follow-up as per the Fleisher’s Society guidelines. The final diagnosis in primary malignant lesions was arrived at based on histopathological diagnosis, and metastasis was based on the histopathological diagnosis of the lung nodule or primary tumor. Only patients in whom no other synchronous lung pathology was identified were included to prevent overlap of pathologies. Patients with subsolid pulmonary nodules and Nodules with calcification were excluded from the study to compare purely solid pulmonary nodules. Of the other benign lesions (*n* = 39) analyzed using radiomics, 36% were septic emboli, 28%were benign lesions monitored long-term per Fleishner society guidelines, and the remainder comprised sarcoidosis, inflammatory conditions, pulmonary tuberculosis, benign carcinoid, hematoma, hydatid disease, Sjogren’s syndrome, and Wegner’s granulomatosis cases, as shown in Supplementary Information (Fig. S1). Among the malignant nodules (*n* = 49), there were 25 cases of primary lung malignancies and 24 cases of metastases. Primary lung malignancies consisted of adenocarcinoma (52%), squamous cell carcinoma (40%), and other types, as depicted in Supplementary Information (Fig. S2). The predominant source of metastases was breast carcinoma, with the remainder originating from various other primary organs, as depicted in Supplementary Information (Fig. S3).

### CT acquisition

HRCT examinations were performed using one of the following multidetector computed tomography (MDCT) scanners: Phillips-brilliance 16 (Philips medical systems, Cleveland); GE EVO evolution 128 slices (GE healthcare, Princeton); and Siemens biograph horizon (Siemens AG, Munich). HR-CT images were obtained during breath-holding with the following parameters: 120 kV, 200 mA. The section thickness and reconstruction intervals were 0.65–0.80 mm. The CT images were sent to a picture archiving and communication system (PACS) to be interpreted at workstations.

### Segmentation

**Figure 3:**
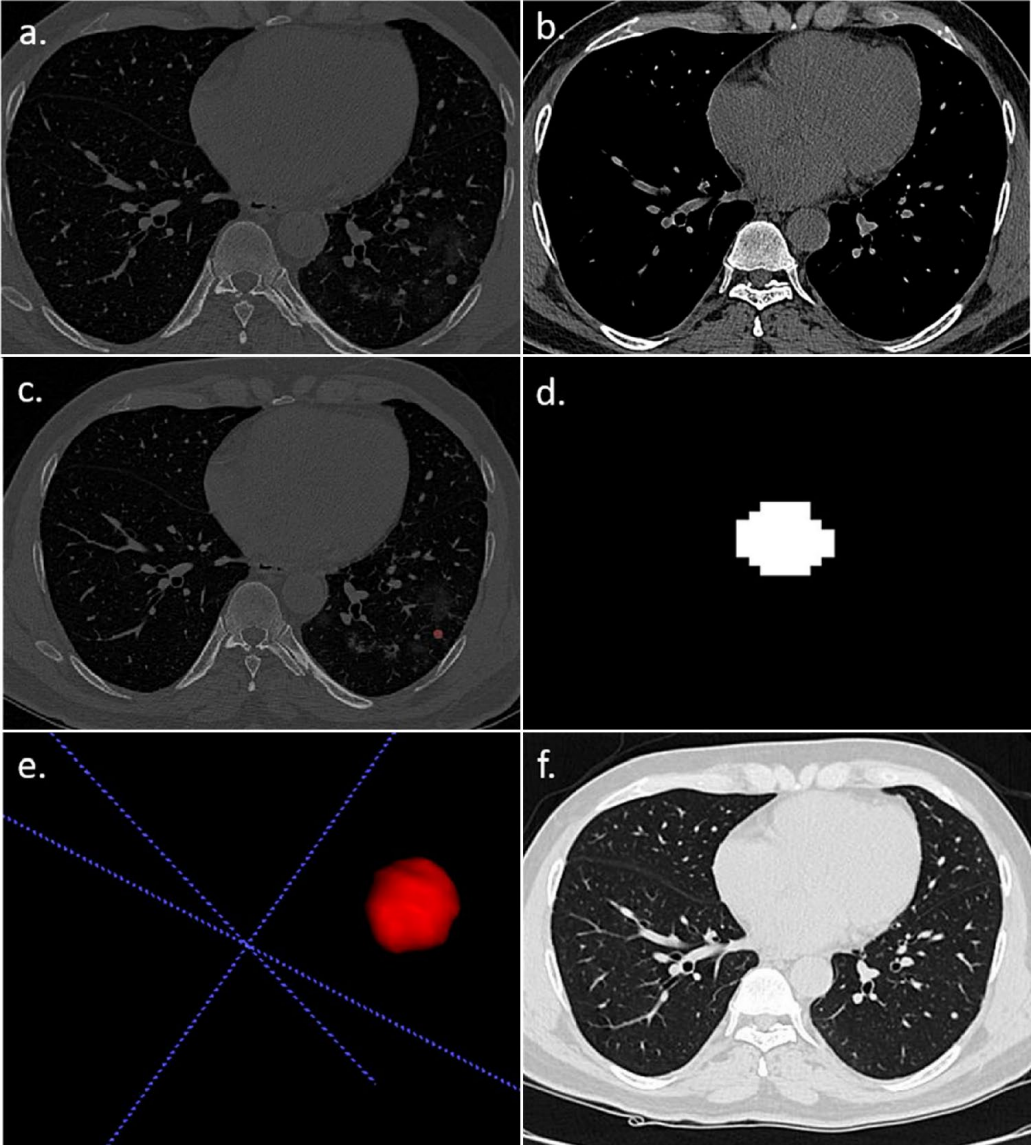
A 57 year-old male patient with RT–PCR has proven COVID-19 pneumonia. (**a**,**b**) The axial section of the CT thorax in the lung window and soft tissue window shows a subpleural soft tissue nodule in the posterior segment of the left lower lobe. (**c**) Creation of ROI for segmentation. (**d**) 2D-segmented nodule. (**e**) 3D-volumetric rendering of the nodule. (**f**) Follow-up chest CT after 6 months revealed partial resolution of the nodule.

**Figure 4:**
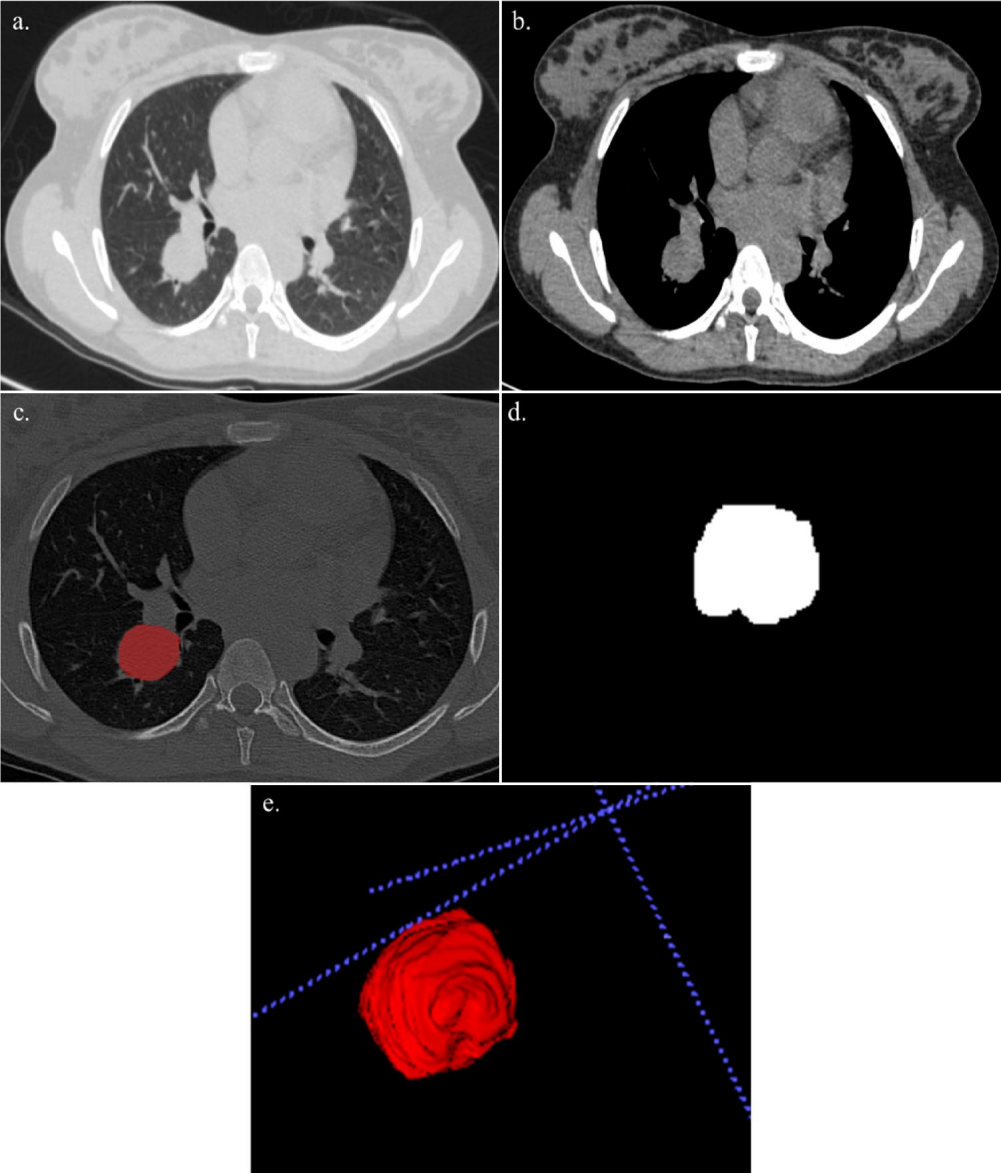
A 21 year-old lady with cough and hemoptysis. HPE: benign carcinoid tumor. (**a**,**b**) Axial section of CT thorax in lung window and soft tissue window showing a mass lesion in the posterior segment of the right lower lobe. (**c**) Creation of ROI for segmentation. (**d**) 2D segmented mass lesion. (**e**) 3D volumetric rendering of the mass lesion.

The segmentation of the DICOM images of the pulmonary nodules, a critical initial step for accurate feature extraction, was performed manually by an expert radiologist using Insight Segmentation and Registration Toolkit (ITK-SNAP) software^19^ and was verified by three radiologists independently. The steps described above are shown in Figs. 3 and 4. By relying on the expert radiologist, we could delineate the nodules with a high degree of precision, particularly in terms of their shape and texture characteristics, which are crucial for subsequent radiomic analysis. Following the segmentation, we extracted radiomic features from the 3D representations of the nodules. The extraction process focused on a comprehensive set of features, including but not limited to, shape, size, intensity, texture, and wavelet features. The emphasis was on capturing a broad spectrum of information that reflects the underlying pathology and can be correlated with clinical outcomes. By combining expert radiological input with radiomic feature extraction techniques, we aimed to mitigate some of the challenges associated with parametric texture feature extraction.

### Radiomics analysis

**Figure 5:**
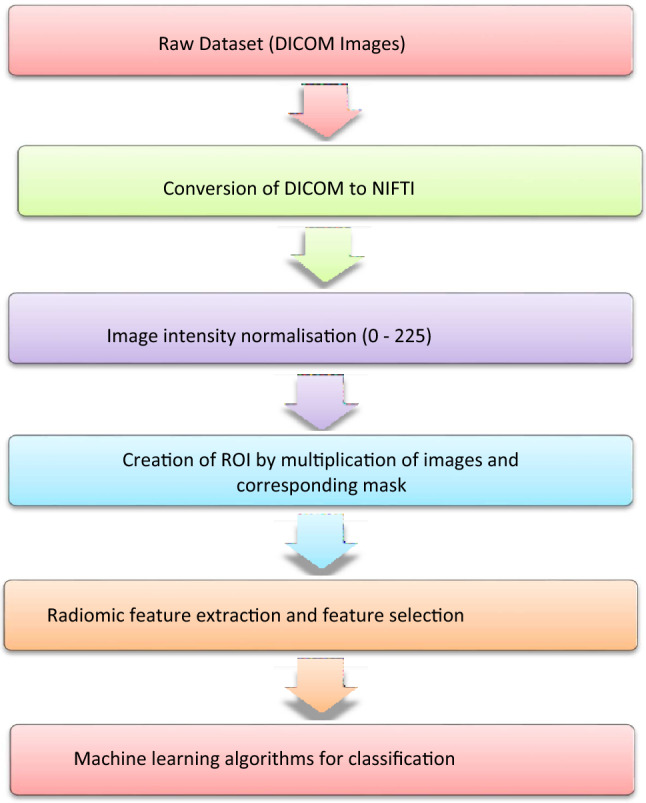
Radiomics workflow.

**Figure 6:**
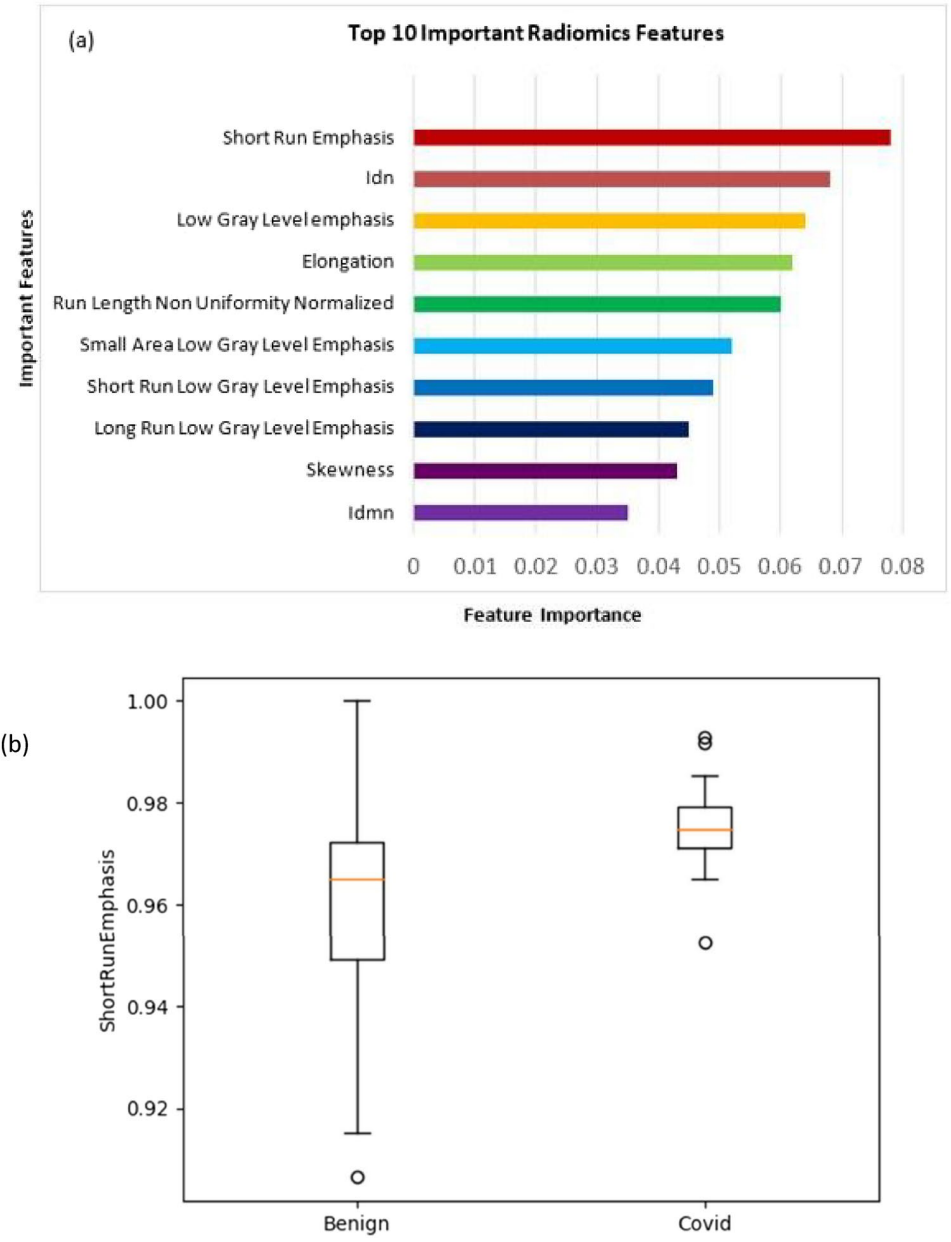
(**a**) Ten important features used for the classification of COVID-19 and non-COVID-19 benign lung nodules. (**b**) Comparison plot of the most prominent feature.

Segmented lung nodules were used to extract different types of features. These features were classified into three categories: shape features (14), first-order features (18 features), and texture-based features (69 features). Texture-based features were of four types, namely gray level co-occurrence matrix (GLCM) features (24 features)^20^, gray-level run-length matrix (GLRLM) features (16 features)^21^, gray level size zone (GLSZM) features (16 features)^15,22,23^ and gray level dependence matrix (GLDM) features (13 features). Each radiomics feature was given a feature rank based on a random forest classifier. Out of 101, the top 10 features were selected for classification algorithms according to Anand et al.^24^. Figures 5 and 6 show the top 10 selected radiomics features with rank, and Tables 1 and 2 summarize their feature importance values. Several classification algorithms, such as SUPPORT VECTOR MACHine (SVM)^25^, multi-layer perceptron (MLP), naive Bayes, discriminant analysis, and decision tree^26^, were applied to selected feature matrices to classify benign and malignant nodules. SVM with the linear kernel (L-SVM) and radial basis function kernel (RBF–SVM) were used as SVM variants. Linear discriminant analysis (LDA) and quadratic discriminant analysis (QDA) were used in the category of discriminant analysis. We have also experimented with MLP classifiers for different hyperparameters which include activation, layers/number of neurons, and learning rate. To evaluate the performance of classifiers, confusion matrices were drawn on the test set. Accuracy, sensitivity, specificity, precision, and F1-measure were calculated for each classifier.

**Table 1:** COVID-19 vs non-COVID-19 Benign lung nodules-different performance metrics for different classifiers obtained on the test data set.

Classifiers	Accuracy	Sensitivity	Specificity	Precision	F1_score
Nearest neighbours	0.693	0.6	0.750	0.60	0.60
Linear SVM	0.643	0.2	0.889	0.50	0.29
RBF SVM	0.615	0.0	1.000	0.00	0.00
Decision tree	0.692	0.6	0.750	0.60	0.60
Random forest	0.714	0.6	0.778	0.60	0.60
AdaBoost	0.714	0.6	0.778	0.60	0.60
Naive Bayes	0.643	0.8	0.556	0.50	0.62
LDA	0.714	0.4	0.889	0.67	0.50
QDA	0.769	0.6	0.875	0.75	0.67
MLP classifier	**0.83**	**0.83**	**0.88**	**0.83**	**0.83**

Significant values are given in bold.

**Table 2:** Experiment 2: COVID-19 vs malignant lung nodules-different performance metrics for different classifiers obtained on the test data set.

Classifiers	Accuracy	Sensitivity	Specificity	Precision	F1_score
Nearest neighbours	0.688	0.40	0.82	0.50	0.44
Linear SVM	0.733	0.20	1.00	1.00	0.33
RBF SVM	0.650	0.17	0.91	0.50	0.25
Decision tree	**0.750**	**0.60**	**0.82**	**0.60**	**0.60**
Random forest	0.688	0.40	0.82	0.50	0.44
AdaBoost	0.650	0.50	0.73	0.50	0.50
Naive Bayes	0.625	0.80	0.55	0.44	0.57
LDA	**0.800**	**0.60**	**0.90**	**0.75**	**0.67**
QDA	0.670	0.40	0.80	0.50	0.44
MLP classifier	**0.86**	**0.86**	**0.90**	**0.86**	**0.86**

Significant values are given in bold.

While many state-of-the-art approaches in medical image analysis today do use deep learning methods, in our experiments they showed poor performance with an accuracy of at most 55%. We evaluated models such as ResNet, DenseNet, and Vision Transformer for the same but due to the limited data available, the models showed poor performance^27^. The radiomic features provide a more robust basis for training on limited data as compared to the deep learning approaches.

### Ethical clearance

The study was performed after obtaining prior approval from the Institutional Research Ethics Committee—Sri Ramachandra Institute of Higher Education and Research (CSP–MED/19/SEP/56/122) and all methods were performed by relevant guidelines and regulations.

### Informed consent

Informed consent was obtained from all subjects and/or their legal guardians involved in the study.

## Results

From our dataset of 108 cases, 20 were lung nodules in COVID-19 pneumonia, 39 cases were benign non-COVID-19, and 49 were malignant nodules. The workflow of the experiment is shown in Fig. 5. The data was split into the train set and test set in the ratio of 80:20. Classifiers were trained on training sets, and test sets were used to check the performance of classifiers on unseen data sets. Data sets were shuffled and split randomly. In this fashion, five data sets were created and used for training and testing classifiers. The performance of the classifiers was obtained from the confusion matrix. Accuracy, sensitivity, specificity, precision, and F1-score have been used as metrics for different classifiers. The mean of different matrices was taken. Confusion matrices were obtained on the train and test data set. Earlier, we conducted a similar radiomics-based model aimed at distinguishing between benign and malignant pulmonary nodules^28,29^.

### COVID-19 vs non-COVID-19 benign lung nodules

Our study showed that the top 10 features of importance (in decreasing order of importance; Fig. 6a) in differentiating between COVID-19 and other benign non-COVID-19 lung nodules were short run emphasis (SRE), inverse difference normalized (IDN), low gray level emphasis (LGLE), elongation, run length non-uniformity normalized (RLNN), small area low gray level emphasis (SALGLE), short run low gray level emphasis (SRLGLE), long run low gray level emphasis (LRLGLE), skewness, and inverse difference moment normalized (IDMN). After experimenting with different hyperparameters in MLP Classifier few hyperparameter combinations gave the best result. We got an accuracy of 83% on this dataset (Supplementary Information; Table S1). The sensitivity and specificity of our model were 83% and 88%, respectively whereas our model’s precision and F1 score were 83% and 83%, respectively, as shown in Table 1.

### COVID-19 vs malignant lung nodules

We observed that the top 10 features of importance (in decreasing order of importance; Fig. 6a) in differentiating between COVID-19 and malignant lung nodules were root mean squared (RMS), mesh volume, zone percentage (ZP), low gray level emphasis (LGLE), short run low gray level emphasis (SRLGLE), maximum 2D diameter (column), cluster prominence, short run emphasis (SRE), large area emphasis (LAE), and maximum probability. We experimented with different models as well as did a hyperparameter search on the MLP Classifier and the MLP Classifier with tanh activation with a learning rate of 0.01 gave the best result. We got an accuracy of 86% on this dataset. (Supplementary Information; Table S2) The sensitivity and specificity of our model were 86% and 90%, respectively, whereas the precision and F1 scores of our model were 86% and 86%, respectively, as shown in Table 2.

## Discussion

We analyzed HR-CT thorax scan findings in 250 patients with RT–PCR proved COVID-19 infection. All CT scans were done between the 3rd to 10th day of the onset of symptoms. Lung nodules were identified in 24 of these patients (9.6 %), of which, four cases were excluded as radiomics analysis could not be performed on them due to technical issues. The age distribution of our study participants ranged from 24–75 years with the largest number seen in the 5th decade of life. 55 % of our cases were female patients and the remainder were male patients. Solitary nodules were seen in seven cases and multiple nodules in 13 cases. The majority of these nodules were subpleural in distribution (*n* = 17) with the rest being centrilobular (*n* = 2) or perifissural (*n* = 1). All nodules were well-defined and ranged in size from 2 to 13 mm with a mean diameter of 5 mm. They were predominantly located in the left lower lobe (35%) followed by the right upper lobe (25 %) left upper lobe (20%) and the right middle lobe (20%) (*n* = 14) cases were solid nodules and the rest (*n* = 6) were partly solid. In addition to nodules, ground-glass opacities were seen in all 20 patients, consolidation in four, and crazy paving in two patients. The CT COVID-19 reporting and data system (CO-RADS) score was CORADS-6 (RT–PCR-proven cases). The radiomics analysis of these lung nodules in COVID-19 cases was compared with the benign and malignant lung nodules of similar sizes.

For this study, we used a dataset of 108 lung nodules of 3–30 mm size of which 20 were lung nodules in COVID-19 pneumonia, 39 were benign (non-COVID-19) lung nodules, 49 were malignant lung nodules, and the remainder were COVID-19 nodules from RT-PCR proved cases of COVID-19 pneumonia and two different experiments were performed. In the first experiment, the radiomics features of COVID-19 lung nodules and other non-benign lung nodules were compared and in the second experiment, the Radiomics features of COVID-19 lung nodules and malignant lung nodules were compared. In each of these experiments, the data was split into the train set and test set in the ratio of 80:20. Classifiers were trained on training sets, and test sets were used to check the performance of classifiers on unseen data sets. Data sets were shuffled and split randomly. In this fashion, five sets of data were created and used for training and testing classifiers. The performance of the classifiers was obtained from the confusion matrices. Accuracy, sensitivity, specificity, precision, and F1-score were used as metrics for different classifiers. The mean of different metrics has been taken. Confusion matrices were obtained on the train and test data set. These are shown in Figs. 5 and 6. Radiomics analysis was done separately for COVID-19 lung nodules and compared with non-COVID-19 benign lung nodules and malignant lung nodules.

Our study showed that the top 10 features of importance (in decreasing order of importance) in differentiating between COVID-19 and other benign non-COVID-19 lung nodules were SRE, IDN, LGLE, elongation, RLNN, SALGLE, SRLGLE, LRLGLE, skewness, and IDMN. After experimenting with different hyperparameters in MLP Classifier few hyperparameter combinations gave the best result. We got an accuracy of 83% on this dataset. The sensitivity and specificity of our model were 83% and 88%, respectively whereas our model's precision and F1 score were 83% and 83%, respectively, as shown in Table 1. A list of important features in distinguishing COVID-19 lung nodules and other benign lung nodules was extracted. Figure 6 shows different features according to their ranks. Similarly, a separate comparative radiomics analysis was done separately for COVID-19 lung nodules and compared with malignant lung nodules. We observed that the top 10 features of importance (in decreasing order of importance) in differentiating between COVID-19 and malignant lung nodules were RMS, mesh volume, ZP, LGLE, short run low gray (SRLGLE), column, cluster prominence, SRE, LAE, and maximum probability.

**Figure 7:**
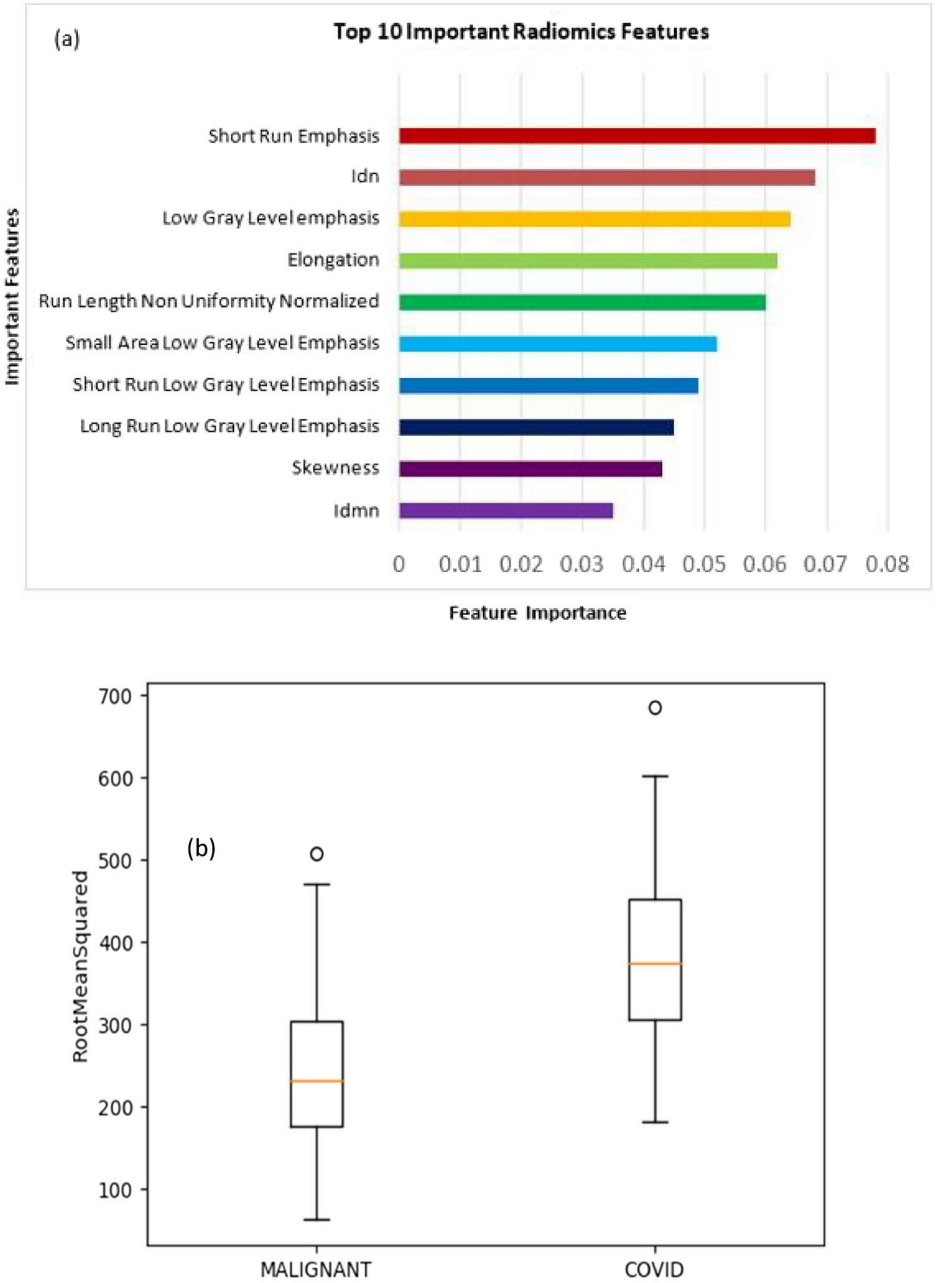
(**a**) Ten important features used for the classification of COVID-19 and malignant lung nodules. (**b**) Comparison plot of most prominent features.

We experimented with different models as well as did a hyperparameter search on the MLP classifier and the MLP Classifier with tanh activation with a learning rate of 0.01 gave the best result. We got an accuracy of 86% on this dataset. Our model’s sensitivity, specificity, precision, and F1 score were 86%, 90%, 86%, and 86%, respectively, as shown in Table 2. A list of important features in distinguishing COVID-19 lung nodules and malignant lung nodules was extracted. Figure 7 shows different features according to their ranks. Figures 3 and 4 illustrate the representative cases of the data. It is imperative to recognize that the dataset employed in our experiments is relatively small since the incidence covid nodules are relatively rare^14^. This limitation necessitates a cautious interpretation of our results. The restricted dataset size may compromise the statistical robustness of our most effective model. However, the methodology and workflow we’ve established remain valuable. They can be further validated and possibly refined through application to larger, more diverse datasets and by conducting multicentric trials. Such steps would help in assessing the generalizability and reliability of our findings across broader contexts.

We had taken the random forest classifier, as it was giving pertinent features. These top 10 features are crucial for distinguishing between COVID and non-benign pulmonary nodules, as well as between COVID and malignant pulmonary nodules, highlighting predominantly variations in density and texture. Rather than assessing individual pixel variations in Hounsfield units (HU), these metrics evaluate the spatial distribution of areas with specific densities (as indicated by HU values). This approach offers clinical significance by providing insights into the overall tissue organization and characteristics that are not visible to the naked eye. By identifying regions with similar densities and analyzing the texture based on the size and intensity of these regions, these features offer valuable information about macroscopic tissue patterns, aiding in clinical decision-making, radiological diagnosis, and treatment planning.

## Conclusion

Lung nodules, though less commonly seen, are one of the imaging manifestations of coronavirus infection. They are generally small in size and subpleural in location. We developed a radiomics model that showed potential as a noninvasive diagnostic method for accurately differentiating COVID-19 lung nodules from benign non-COVID-19 lung nodules and malignant lung nodules. Our study showed that the top 10 features of importance (in decreasing order of importance) in differentiating between COVID-19 and other benign non-COVID-19 lung nodules were SRE, IDN, LGLE, elongation, RLNN, SALGLE, SRLGLE, LRLGLE, Skewness, and IDMN. Of the various classifiers that were used for machine learning algorithms, after experimenting with different hyperparameters in MLP Classifier few hyperparameter combinations gave the best result. We got an accuracy of 83% on this dataset. The sensitivity, specificity, precision, and F1 score of our study were 83%, 88%, 83%, and 83% respectively in differentiating between COVID-19 and other benign non-COVID-19 lung nodules.

We have also observed that the top 10 features of importance (in decreasing order of importance) in differentiating between COVID-19 and malignant lung nodules were RMS, mesh volume, ZP, LGLE, short run low gray (SRLGLE), column, cluster prominence, SRE, LAE, and maximum probability. We experimented with different models as well as did a hyperparameter search on the MLP classifier and the MLP classifier with tanh activation with a learning rate of 0.01 gave the best result. We got an accuracy of 86% on this dataset. Our model's sensitivity, specificity, precision, and F1 score were 86%, 90%, 86%, and 86%, respectively, in differentiating between COVID-19 and malignant lung nodules.

In summary, radiomics can be used as a powerful tool in modern medicine, as an adjunct to CT and/or PET–CT images, in further evaluation of lung nodules, and to help in differentiating between COVID-19 lung nodules and other benign non-COVID-19 and malignant nodules. This in turn would help in clinical decision-making and improve diagnostic, predictive, and prognostic accuracy. Radiomics, unlike biopsies, is non-invasive, three-dimensional, and provides information regarding the entire nodule. It also helps in reducing the number of benign biopsies. With more multicentric trials and standardization, radiomics shortly will have an important complementary role in lung nodule evaluation, and diagnosis which can aid in selecting patients for biopsies and guiding treatment options.

### Supplementary Information

Supplementary Information.

## Data Availability

The datasets generated or analyzed during the study are available from the corresponding author upon reasonable request.

## Abbreviations

COVID-19: Corona virus disease of 2019
CO-RADS: COVID-19 Reporting and Data System
CT: Computed tomography
HR-CT: High-resolution computed tomography
DICOM: Digital imaging and communications in medicine
GGO: Ground-glass opacities
GLCM: Gray level co-occurrence matrix
GLDM: Gray level dependence matrix
GLRLM: Gray-level run-length matrix
GLSZM: Gray level size zone
HU: Hounsfield units
ITK-SNAP: Insight segmentation and registration toolkit
IDN: Inverse difference normalized
IDMN: Inverse difference moment normalized
LAE: Large area emphasis
LDA: Linear discriminant analysis
L-SVM: Support vector machine with linear kernel
LGLE: Low gray level emphasis
LRLGLE: Long run low gray level emphasis
LGLE: Low gray level emphasis
MD-CT: Multidetector computed tomography
MLP: Multi-layer perceptron
PACS: Picture archiving and communication system
PET-CT: Positron emission tomography and computed tomography
QDA: Quadratic discriminant analysis
RBF-SVM: Support vector machine with radial basis function kernel
ReLU: Rectified linear unit
RLNN: Run length non-uniformity normalized
RMS: Root mean squared
RT-PCR: Reverse transcription-polymerase chain reaction
SALGLE: Small area low gray level emphasis
SARS-CoV-2: Severe acute respiratory syndrome coronavirus-2
SRE: Short run emphasis
SRLGLE: Short run low gray level emphasis
SVM: Support vector machine
WHO: World Health Organization
ZP: Zone percentage
