# Supplementary material for: Radiomics analysis for distinctive identification of COVID-19 pulmonary nodules from other benign and malignant counterparts
Source: Sci Rep. 2024 Mar 25;14:7079. doi: 10.1038/s41598-024-57899-x (PMC10963772; doi:10.1038/s41598-024-57899-x)
Supplement: Supplementary file 1 — Supplementary Information. [file 41598_2024_57899_MOESM1_ESM.pdf]

Supplementary Information

Radiomics analysis for distinctive identification of COVID pulmonary nodules from other benign and malignant counterparts

Minmini Selvam,[1*] Anupama Chandrasekharan,[1] Abjasree Sadanandan,[2] Vikas K. Anand,[2] Sidharth Ramesh,[2] Arunan Murali[1], Ganapathy Krishnamurthi[2]

[1] Department of Radiology and Imaging Sciences, Sri Ramachandra Institute of Higher Education and Research, Porur, Chennai 600 116, India

[2] Department of Engineering Design, Indian Institute of Technology-Madras, Chennai 600 036, India

[*]**Corresponding Author**

Minmini Selvam, MD, DNB, MICR, EDiR

Department of Radiology and Imaging Sciences
Sri Ramachandra Institute of Higher Education and Research
Porur, Chennai 600 116, India
Tel: 91-9444-579-122
E-mail: sminmini@yahoo.co.in

Figures and Tables

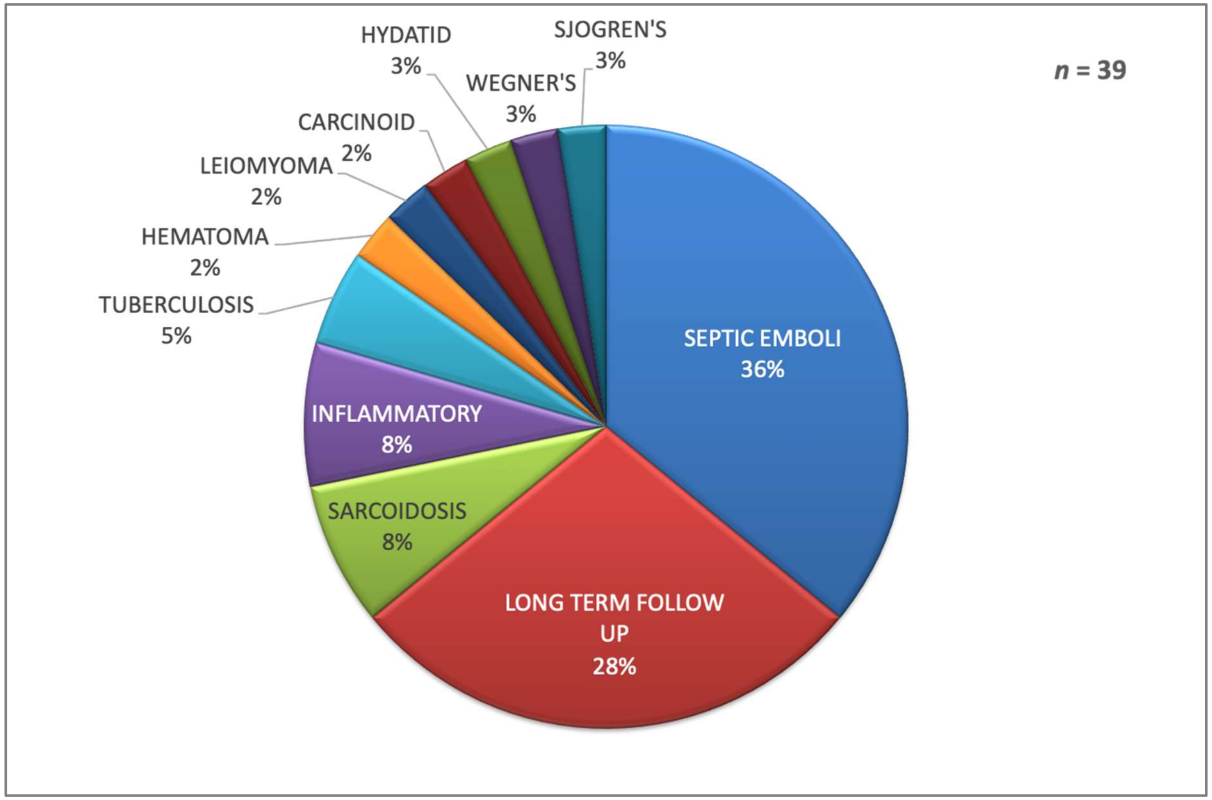

Figure S1. Case distribution of other benign lung nodules.

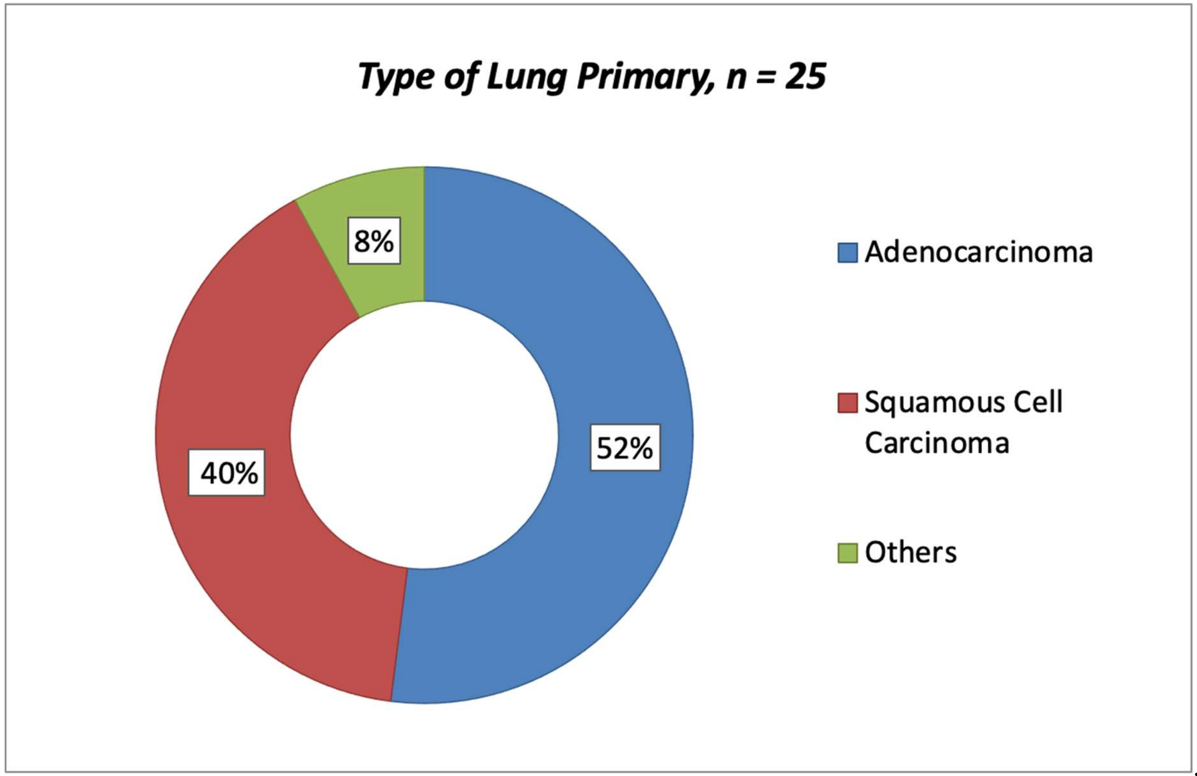

Figure S2. Case distribution of primary lung malignancy presenting as lung nodules.

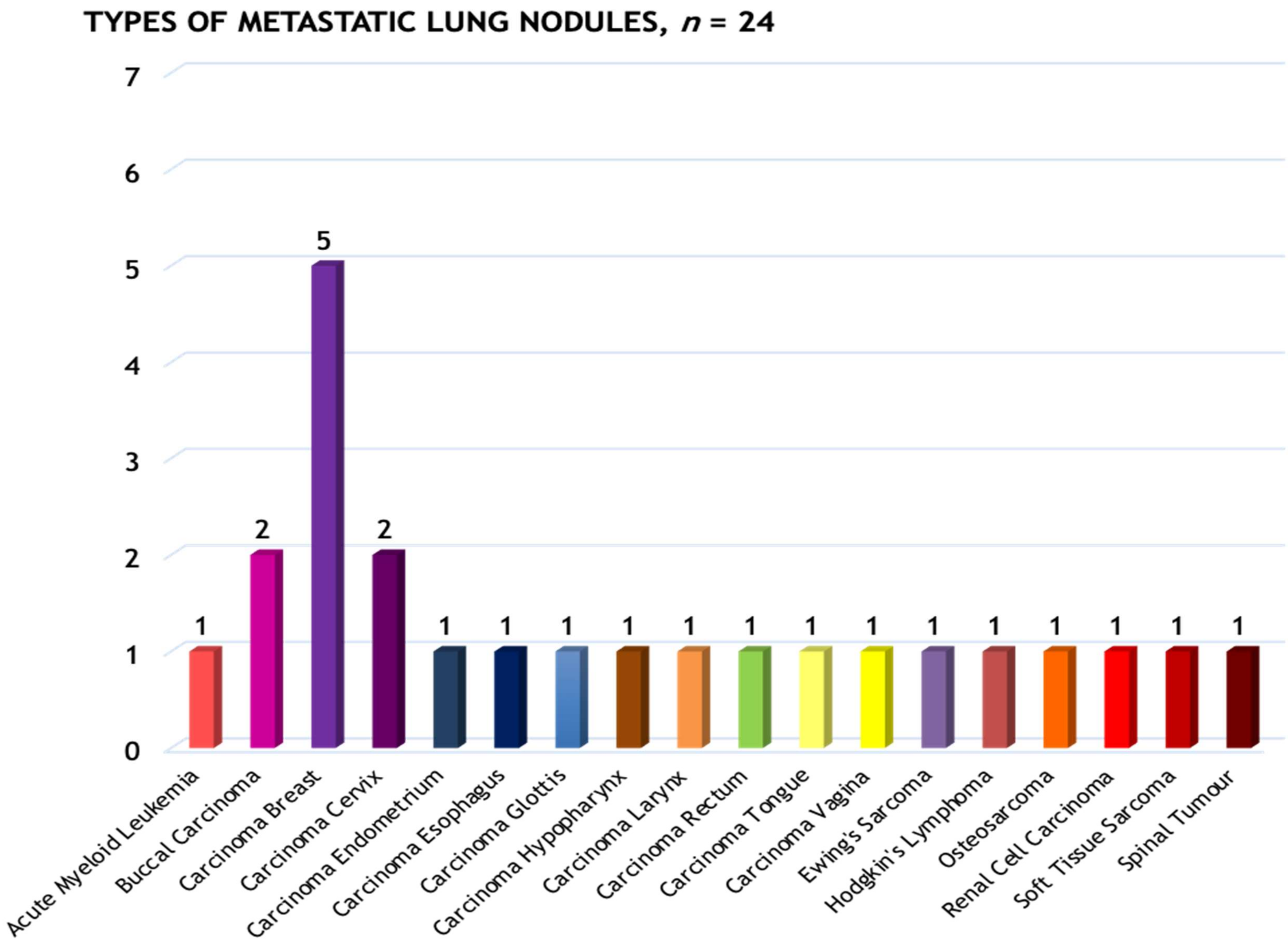

Figure S3. Case distribution of metastatic pulmonary nodules.

Table S1. The hyperparameter tuning experimental results for the benign-covid classification.

Activation	Hidden layer sizes	Learning rate	Precision	Recall	Accuracy	F1 score	Specificity
identity	50	0.001	0.67	0.67	0.67	0.67	0.75
identity	50	0.01	0.77	0.75	0.75	0.76	0.75
identity	50	0.05	0.67	0.67	0.67	0.67	0.75
identity	100	0.001	0.77	0.75	0.75	0.76	0.75
identity	100	0.01	0.77	0.75	0.75	0.76	0.75
identity	100	0.05	0.67	0.67	0.67	0.67	0.75
identity	100, 50	0.001	0.77	0.75	0.75	0.76	0.75
identity	100, 50	0.01	0.67	0.67	0.67	0.67	0.75
identity	100, 50	0.05	0.77	0.75	0.75	0.76	0.75
identity	150	0.001	0.77	0.75	0.75	0.76	0.75
identity	150	0.01	0.67	0.67	0.67	0.67	0.75
identity	150	0.05	0.67	0.67	0.67	0.67	0.75
logistic	50	0.001	0.77	0.75	0.75	0.76	0.75
logistic	50	0.01	0.83	0.83	0.83	0.83	0.88
logistic	50	0.05	0.83	0.83	0.83	0.83	0.88
logistic	100	0.001	0.77	0.75	0.75	0.76	0.75
logistic	100	0.01	0.77	0.75	0.75	0.76	0.75
logistic	100	0.05	0.83	0.83	0.83	0.83	0.88
logistic	100, 50	0.001	0.77	0.75	0.75	0.76	0.75
logistic	100, 50	0.01	0.83	0.83	0.83	0.83	0.88
logistic	100, 50	0.05	0.83	0.83	0.83	0.83	0.88
logistic	150	0.001	0.67	0.67	0.67	0.67	0.75
logistic	150	0.01	0.83	0.83	0.83	0.83	0.88
logistic	150	0.05	0.83	0.83	0.83	0.83	0.88
tanh	50	0.001	0.77	0.75	0.75	0.76	0.75

tanh	50	0.01	0.83	0.83	0.83	0.83	0.88
tanh	50	0.05	0.83	0.83	0.83	0.83	0.88
tanh	100	0.001	0.77	0.75	0.75	0.76	0.75
tanh	100	0.01	0.83	0.83	0.83	0.83	0.88
tanh	100	0.05	0.83	0.83	0.83	0.83	0.88
tanh	100, 50	0.001	0.77	0.75	0.75	0.76	0.75
tanh	100, 50	0.01	0.83	0.83	0.83	0.83	0.88
tanh	100, 50	0.05	0.77	0.75	0.75	0.76	0.75
tanh	150	0.001	0.77	0.75	0.75	0.76	0.75
tanh	150	0.01	0.83	0.83	0.83	0.83	0.88
tanh	150	0.05	0.77	0.75	0.75	0.76	0.75
relu	50	0.001	0.77	0.75	0.75	0.76	0.75
relu	50	0.01	0.74	0.75	0.75	0.74	0.88
relu	50	0.05	0.74	0.75	0.75	0.74	0.88
relu	100	0.001	0.77	0.75	0.75	0.76	0.75
relu	100	0.01	0.77	0.75	0.75	0.76	0.75
relu	100	0.05	0.67	0.67	0.67	0.67	0.75
relu	100, 50	0.001	0.77	0.75	0.75	0.76	0.75
relu	100, 50	0.01	0.83	0.83	0.83	0.83	0.88
relu	100, 50	0.05	0.83	0.83	0.83	0.83	0.88
relu	150	0.001	0.77	0.75	0.75	0.76	0.75
relu	150	0.01	0.77	0.75	0.75	0.76	0.75
relu	150	0.05	0.83	0.83	0.83	0.83	0.88

Table S2. The hyperparameter tuning experimental results for the malignant-covid classification.

Activation	Hidden layer sizes	Learning rate	Precision	Recall	Accuracy	F1 score	Specificity
identity	50	0.001	0.68	0.71	0.71	0.68	0.9
identity	50	0.01	0.57	0.57	0.57	0.57	0.7
identity	50	0.05	0.45	0.5	0.5	0.48	0.7
identity	100	0.001	0.68	0.71	0.71	0.68	0.9
identity	100	0.01	0.57	0.57	0.57	0.57	0.7
identity	100	0.05	0.45	0.5	0.5	0.48	0.7
identity	100, 50	0.001	0.61	0.64	0.64	0.63	0.8
identity	100, 50	0.01	0.57	0.57	0.57	0.57	0.7
identity	100, 50	0.05	0.68	0.71	0.71	0.68	0.9
identity	150	0.001	0.68	0.71	0.71	0.68	0.9
identity	150	0.01	0.57	0.57	0.57	0.57	0.7
identity	150	0.05	0.45	0.5	0.5	0.48	0.7
logistic	50	0.001	0.61	0.64	0.64	0.63	0.8
logistic	50	0.01	0.61	0.64	0.64	0.63	0.8
logistic	50	0.05	0.77	0.79	0.79	0.78	0.9
logistic	100	0.001	0.68	0.71	0.71	0.68	0.9
logistic	100	0.01	0.77	0.79	0.79	0.78	0.9
logistic	100	0.05	0.77	0.79	0.79	0.78	0.9
logistic	100, 50	0.001	0.68	0.71	0.71	0.68	0.9
logistic	100, 50	0.01	0.77	0.79	0.79	0.78	0.9
logistic	100, 50	0.05	0.88	0.86	0.86	0.84	1
logistic	150	0.001	0.61	0.64	0.64	0.63	0.8
logistic	150	0.01	0.77	0.79	0.79	0.78	0.9
logistic	150	0.05	0.71	0.71	0.71	0.71	0.8
tanh	50	0.001	0.77	0.79	0.79	0.78	0.9
tanh	50	0.01	0.77	0.71	0.71	0.73	0.7

tanh	50	0.05	0.81	0.79	0.79	0.79	0.8
tanh	100	0.001	0.68	0.71	0.71	0.68	0.9
tanh	100	0.01	0.86	0.86	0.86	0.86	0.9
tanh	100	0.05	0.77	0.71	0.71	0.73	0.7
tanh	100, 50	0.001	0.77	0.79	0.79	0.78	0.9
tanh	100, 50	0.01	0.81	0.79	0.79	0.79	0.8
tanh	100, 50	0.05	0.68	0.71	0.71	0.68	0.9
tanh	150	0.001	0.68	0.71	0.71	0.68	0.9
tanh	150	0.01	0.86	0.86	0.86	0.86	0.9
tanh	150	0.05	0.81	0.79	0.79	0.79	0.8
relu	50	0.001	0.68	0.71	0.71	0.68	0.9
relu	50	0.01	0.77	0.79	0.79	0.78	0.9
relu	50	0.05	0.77	0.79	0.79	0.78	0.9
relu	100	0.001	0.68	0.71	0.71	0.68	0.9
relu	100	0.01	0.77	0.79	0.79	0.78	0.9
relu	100	0.05	0.71	0.71	0.71	0.71	0.8
relu	100, 50	0.001	0.77	0.79	0.79	0.78	0.9
relu	100, 50	0.01	0.77	0.79	0.79	0.78	0.9
relu	100, 50	0.05	0.71	0.71	0.71	0.71	0.8
relu	150	0.001	0.77	0.79	0.79	0.78	0.9
relu	150	0.01	0.77	0.79	0.79	0.78	0.9
relu	150	0.05	0.77	0.79	0.79	0.78	0.9
